# Dietary iron depletion at weaning imprints low microbiome diversity and this is not recovered with oral nano Fe(III)

**DOI:** 10.1002/mbo3.213

**Published:** 2014-12-02

**Authors:** Dora I A Pereira, Mohamad F Aslam, David M Frazer, Annemarie Schmidt, Gemma E Walton, Anne L McCartney, Glenn R Gibson, Greg J Anderson, Jonathan J Powell

**Affiliations:** 1 MRC Human Nutrition Research, Elsie Widdowson LaboratoryCambridge, United Kingdom; 2 Iron Metabolism Laboratory, QIMR Berghofer Medical Research InstituteBrisbane, Australia; 3 Department of Food and Nutritional Sciences, University of ReadingReading, United Kingdom

**Keywords:** Iron supplementation, microbiome, microbiota, nanoparticles, oral iron

## Abstract

Alterations in the gut microbiota have been recently linked to oral iron. We conducted two feeding studies including an initial diet-induced iron-depletion period followed by supplementation with nanoparticulate tartrate-modified ferrihydrite (Nano Fe(III): considered bioavailable to host but not bacteria) or soluble ferrous sulfate (FeSO_4_: considered bioavailable to both host and bacteria). We applied denaturing gradient gel electrophoresis and fluorescence in situ hybridization for study-1 and 454-pyrosequencing of fecal 16S rRNA in study-2. In study-1, the within-community microbial diversity increased with FeSO_4_ (*P* = 0.0009) but not with Nano Fe(III) supplementation. This was confirmed in study-2, where we also showed that iron depletion at weaning imprinted significantly lower within- and between-community microbial diversity compared to mice weaned onto the iron-sufficient reference diet (*P* < 0.0001). Subsequent supplementation with FeSO_4_ partially restored the within-community diversity (*P* = 0.006 in relation to the continuously iron-depleted group) but not the between-community diversity, whereas Nano Fe(III) had no effect. We conclude that (1) dietary iron depletion at weaning imprints low diversity in the microbiota that is not, subsequently, easily recovered; (2) in the absence of gastrointestinal disease iron supplementation does not negatively impact the microbiota; and (3) Nano Fe(III) is less available to the gut microbiota.

## Introduction

Iron deficiency anemia remains the largest nutritional disorder worldwide, affecting 1 billion people (WHO 2008). Standard treatment is supplementation with ferrous iron salts (Cook 2005), that are cheap and well absorbed but also associated with significant upper and lower gastrointestinal side effects such as nausea, constipation, and abdominal pain (Cancelo-Hidalgo et al. 2013). However, for the past decade or so animal studies in models of gastrointestinal disease have consistently shown that soluble iron can have a detrimental effect on the unhealthy colon, further promoting inflammation or acting as a marked risk factor for colorectal carcinogenesis (Seril et al. 2002; Werner et al. 2011; Radulescu et al. 2012).

There are few reports on the effects of iron supplementation on the composition of the fecal microbiota (Zimmermann et al. 2010; Werner et al. 2011; Dostal et al. 2012), even though certain gut bacteria are likely to contribute to some of the gastrointestinal symptoms associated with oral iron supplementation (Hartley et al. 1992; Benno et al. 1993; Bullock et al. 2004; Ott et al. 2004; Guarner 2005; de Silva et al. 2005; Arthur et al. 2012; Hooper et al. 2012; Nicholson et al. 2012). Indeed, the WHO has said that research into the impact of oral iron supplements on the gut microbiota to elucidate the mechanisms of the adverse effects associated with oral iron is vital (WHO 2007). The only human data available to date are in anemic African children with high hookworm infection rates (Zimmermann et al. 2010). In this work, Zimmerman et al. showed that these children carried an unfavorable burden of fecal *Enterobacter* compared to beneficial *Bifidobacterium* and *Lactobacillus* and that this was further exacerbated following 6-months of iron supplementation with electrolytic iron-fortified biscuits (Zimmermann et al. 2010). Similarly, in a mouse model of Crohn's disease, Werner et al. (2011) have shown that mice on a ferrous sulfate (FeSO_4_)-supplemented diet had an unfavorable gut microbiota profile with decreased numbers of *Bifidobacterium* and increased numbers of the sulfate-reducing bacteria *Desulfovibrio* in comparison to animals on an iron-deficient diet. Our own work, in severely anemic rats with diarrhea and a high fecal prevalence of the potential pathogen *Escherichia fergusonii* (>50%), has suggested that repletion of iron stores with soluble FeSO_4_ yields a shift of the microbiota toward dominance of *Bacteroides*, whereas repletion of iron stores using a nanoparticulate Fe(III) compound (tartrate-modified ferrihydrite) shifts the dominance toward the genus *Lactobacillus* (Pereira et al. 2014). Nano Fe(III)) is readily bioavailable to the host, and is acquired endocytically by the enterocyte and undergoes chelator-induced dissolution in the cell lysosome (Pereira et al. 2013; Powell et al. 2014). In contrast, it does not redox-cycle in the gut lumen (Powell et al. 2014) and is considered to be of limited bioavailability to bacteria (Pereira et al. 2014).

While all of the above refers to the effects of oral iron on fecal bacteria of a compromised host (i.e., with infection or colonic inflammation) there is, to our knowledge, only one report in an iron deficient but otherwise healthy host. Zimmermann's group showed that diet-induced iron depletion in healthy rats causes significant changes in the gut microbiota composition, with decreases in *Roseburia* and *Bacteroides* (Dostal et al. 2012). They have shown that dietary iron repletion with iron that is bioavailable to host and bacteria (namely, soluble FeSO_4_) restores composition of the fecal microbiota of the severely anemic rats to that of iron-replete animals without any obvious detrimental effect (Dostal et al. 2012). Furthermore, iron that is poorly bioavailable to both host and gut bacteria (namely, poorly soluble electrolytic iron) is not able to restore fecal microbiota composition to that of iron-replete animals (Dostal et al. 2012).

The aim of the present work was to follow on from these findings and to investigate the effect of oral iron that is bioavailable to host but not soluble in the gut lumen and, therefore, presumed poorly available to gut bacteria, on the diversity of the fecal microbiota. We have used molecular methods, namely Fluorescence in situ hybridization (FISH), DGGE or 454-pyrosequencing, and have taken advantage of samples that were collected from two rodent feeding studies (one in rats and one in mice) with dietary iron supplementation in the form of Nano Fe(III) or FeSO_4_. These studies were primarily carried out to investigate the efficacy of Nano Fe(III) at correcting iron deficiency anemia (Aslam et al. 2014; Powell et al. 2014), but have also provided this opportunity for us to investigate the effects of dietary iron depletion, followed by dietary iron repletion with FeSO_4_, or Nano Fe(III) on the fecal microbiota composition.

## Materials and Methods

### Iron materials

Ferrous sulfate heptahydrate (FeSO_4_) and ferric citrate monohydrate pharma-grade were obtained from Sigma-Aldrich (Dorset, U.K.). The Nano Fe(III) was produced using food grade reagents and following the protocol described by Powell et al. (2014). Briefly, an acidic concentrated stock solution of ferric chloride was added to a solution containing tartaric acid and adipic acids at a molar ratio of Fe:tartrate:adipate = 2:1:1. The initial pH of the mixture was below 2.0, and the iron was fully solubilized as determined by ultrafiltration (3000 Da MWCO; 10,000*g*, 10 min). The pH was then slowly increased through drop-wise addition of a concentrated solution of NaOH with constant agitation until the desired final pH (ca. 7.4) was obtained. The entire mixture was then oven-dried at 45°C for a minimum of 24 h. Unmodified synthetic ferrihydrite (i.e., Fe(III) poly oxo-hydroxide, herein abbreviated as Fh) was produced by adding an acidic concentrated stock solution of ferric chloride to 0.9% (w/v) potassium chloride and increasing pH of the resulting solution to 7.4–8.2 with NaOH. The mixture was centrifuged (6000*g*, 15 min), the supernatant discarded, and the solid phase dried at 45°C for a minimum of 8 h.

### Animal studies

#### First study

The study was conducted by MPI Research (Michigan) and was approved by the Institutional Animal Care and Use Committee (IACUC) (MPI study protocol number 1925-001) following United States Animal Welfare Regulations. Male Sprague–Dawley [Crl:CD® (SD)] rats (*n* = 32) were ∼21 days old at arrival (Charles River Laboratories Portage, MI, U.S.A.). The animals were housed individually in polyboxes with toys and fed Block Lab Diet® Certified Rodent Diet #5002 (PMI Nutrition International, Inc., Shoreview, MN, U.S.A.) ad libitum during the acclimation period (not <7 days). The rats were then switched to the iron-deficient diet (AIN-93G Purified Rodent Diet; Dyets Inc., Bethlehem, PA, U.S.A.) which was administered to all animals from day 0 to day 24 of the study (for the complete diet composition see Table S1). Incorporation of the iron materials into the diets (ca. 35 mg Fe/kg diet) and pelletization through extrusion was carried out by Dyets Inc. Other than the varying iron compound, the diets were equivalent and conformed to AIN-93G purified rodent diet (Reeves et al. 1993).

**Figure 1:**
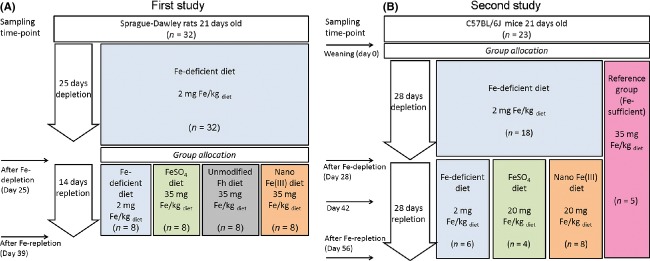
Design of animal studies. (A) feeding study in Fe-deficient Sprague–Dawley rats (Powell et al. 2014). (B) feeding study in Fe-deficient C57BL/6J mice (Aslam et al. 2014). The reference iron-replete group was fed the regular Fe-sufficient diet (i.e., modified AIN-93G purified rodent diet (Aslam et al. 2014), which contains ferric citrate as the iron source) for the entire duration of the study.

Administration of the test diets, iron-deficient diet fortified with either FeSO_4_, unmodified Fh, or Nano Fe(III), began on day 25 of the study as per the outline in Figure1A. Three groups of eight male rats were administered the test diets for 14 days. One additional group of eight animals served as the iron-deficient (control) group and continued to receive the unsupplemented diet for a further 14 days (i.e., until day 38). Rats consumed tap water and food ad libitum throughout the study. Fecal samples for microbiota evaluations were collected on days 25 (prior to changing feed) and 39 of the study. Blood samples were collected on days −1, 25 and 33 for hemoglobin evaluation by clinical pathology as part of a complete blood count (CBC) panel, and these data are reported elsewhere (Powell et al. 2014). After the study termination (day 39), the rats were anaesthetized by CO_2_ inhalation, and animals were euthanized by exsanguination.

#### Second study

The study was conducted and approved by the QIMR Berghofer Medical Research Institute. Twenty-one-day-old male C57BL/6J mice (*n* = 18) were housed individually and fed ad libitum an iron-deficient diet as above for 28 days. Animals had unlimited access to deionized water throughout. Following this iron-depletion period, animals were administered ad libitum one of the two test diets for another 28 days as per study outline in Figure1B. One of the groups of animals (*n* = 4) was administered the iron-deficient diet supplemented with FeSO_4_ (ca. 20 mg Fe/kg diet) and another group (*n* = 8) was administered the iron-deficient diet supplemented with Nano Fe(III) (ca. 20 mg Fe/kg diet). Apart from the iron source, the diets were equivalent and conformed to AIN-93G purified rodent diet (Reeves et al. 1993). One additional group of animals (*n* = 6) remained on the iron-deficient diet throughout the study period (iron-deficient control group). In parallel to this, a reference group of 5 mice was fed ad libitum the standard AIN-93G diet (Reeves et al. 1993) (with ca. 35 mg Fe/kg diet as ferric citrate: here termed Fe-sufficient diet) for the entire study period, and served as the iron-replete reference group. Incorporation of the iron materials into the powder diets was carried out by Speciality Feed (WA, Australia). Fecal samples were collected from each animal on days 0, 28, 42, and 56 for microbiota analysis. Blood samples were collected from each animal on days 0, 28, 35, 42, and 56 for hemoglobin measurements. After the study termination (day 56), mice were anaesthetized with a single intraperitoneal injection of xylazine (8 mg/kg) and ketamine (44 mg/kg), and the mice were euthanized by exsanguination and blood and liver, spleen, and duodenal tissues were collected. Hemoglobin and tissue analysis are reported elsewhere (Aslam et al. 2014).

### Microbiota analysis

#### Fluorescence in situ hybridisation

Fecal pellets were homogenized (2:1) in DPBS (Dulbecco's phosphate buffered saline) and samples fixed with 4% (w/v) paraformaldehyde (PFA) at 4°C for ∼16 h. The samples were then centrifuged at 4°C (15,000*g*, 5 min), washed three times with ice-cold DPBS, and resuspended in an ice-cold mixture of 1:1 DPBS:ethanol. Samples were stored at −20°C until analysis. FISH was carried out following the protocol described by Daims et al. (2005). Briefly, fixed samples were diluted and applied to the well of a 6-well PTFE/poly-l-lysine coated slide (Tekdon Inc., Myakka City, FL). Slides were dried at 50°C, then dehydrated in ascending concentrations of ethanol for 3 min (50%, 80%, 96%) then dried before hybridization buffer containing the relevant probe was applied to the slides.

Group-specific 16S rRNA-targeted oligonucleotide probes labeled with the fluorescent dye Cy3 were used for enumerating *Bacteroides/Prevotella*, *Bifidobacterium*, *Bacillus*, *Desulfovibrio*, *Lactobacillus*/*Enterococcus*, *Roseburia*, and segmented filamentous bacteria (Table S2). Total bacteria were enumerated with the oligonucleotide probe Eub338mix specific for domain bacteria. Hybridization was carried out at the appropriate temperature for each probe (Table S2) for 4 h in a hybridization oven (microarray hybridization incubator; Grant-Boekel, Cambridge, UK). Slides were washed in prewarmed washing buffer, containing 4′,6′-diamino-2-phenylindole (DAPI) DNA dye for 15 min at the appropriate temperature (Table S2). A drop of DABCO, antifade solution (Sigma-Aldrich Company Ltd., Dorset, UK) was applied to the slide, along with a cover slip. Slides were enumerated using an epifluorescence microscope (Eclipse E400; Nikon UK, Kingston upon Thames, UK), with counts conducted on 15 random fields of view. The Cy3 dye was viewed under the DM 575 filter to enable enumeration of the bacterial group of interest. DAPI was viewed under the ultraviolet light filter and served as a counterstain, for further confirmation of the presence of DNA.

#### Denaturing gradient gel electrophoresis

Fecal DNA was extracted using a FastDNA®Spin Kit (MP Biomedicals, Cambridge, UK) according to manufacturer's instructions. The DNA concentration was quantified with a nanodrop spectrophotometer and diluted to 50 ng/*μ*L with autoclaved, filtered HPLC-water (high-performance liquid chromatography grade). Touchdown polymerase chain reaction (PCR) amplification of the V3 region of the 16S rRNA genes was performed with 50 ng of DNA using primers p2 and p3 as described by Muyzer et al. (1993). DGGE was performed using the INGENYphorU2 system (GRI) following the protocol described by Waldram et al. (2009). Briefly, PCR products were applied onto polyacrylamide gels in 0.5X TAE buffer (Fisher Scientific, Loughborough, U.K.) with 30–60% gradient formed with 8% (v/v) acrylamide stock solutions (40% acrylamide/bis solution, 37.5:1 [2.6% C]; Bio-Rad, Hemel Hempstead, UK) containing 2% (v/v) glycerol (BDH, Poole, UK), and which contained 0% and 100% denaturant ([7M PlusOne urea; Amersham Pharmacia Biotech, Little Chalfont, UK] and 40% [w/v] PlusOne formamide [Amersham Pharmacia Biotech]). Electrophoresis was run at constant voltage of 100 V and a temperature of 60°C for 16 h. Following electrophoresis, the gels were silver-stained according to the method of Sanguinetti et al. (1994) with minor modifications. Gels were scanned and analysis was performed using GelCompar (BioNumerics, Applied Maths, Austin, TX, U.S.A.). All 32 samples were run on the same gel.

#### 454-pyrosequencing

Fecal DNA was extracted using a QIAamp DNA Stool Mini Kit (Qiagen, VIC, Australia) as per the manufacturer's protocol and quantified with a nanodrop spectrophotometer. Gut microbiota composition was analyzed by 454-pyrosequencing of the total fecal community 16S rRNA gene. This analysis was performed by Molecular Research LP (www.mrdnalab.com, Shallowater, TX) using a bacterial 16S-based tag–encoded FLX amplicon pyrosequencing (bTEFAP®) method (Dowd et al. 2008b). 16S universal Eubacterial primers (27F, (Frank et al. 2008)) were used and the conditions for the single-cell 30 cycle PCR (HotStarTaq Plus Master Mix Kit; Qiagen, Valencia, CA) were as follows: 3 min at 94°C, 28 cycles of 30 sec at 94°C, 40 sec at 53°C, 1 min at 72°C, and a final elongation step 5 min at 72°C. Following PCR, all amplicon products were mixed in equal concentrations and purified using Agencourt Ampure beads (Agencourt Bioscience Corporation, Beverly, MA, U.S.A.). Samples were sequenced utilizing Roche 454 FLX titanium instruments and reagents, following the manufacturer's protocol.

The sequence data were processed using the Molecular Research LP proprietary analysis pipeline using a quality threshold of Q25. In summary, sequences were depleted of barcodes, primers, and short sequences <200 bp. Sequences with ambiguous base calls and sequences with homopolymer runs exceeding 6 bp were also removed. Sequences were then “denoised” and operational taxonomic units (OTU) were defined by clustering at 3% divergence (97% similarity) followed by removal of singleton sequences and chimeras using a proprietary software (MR DNA, Shallowater, TX) (Dowd et al. 2008a,b, 2011; Edgar 2010; Capone et al. 2011; Eren et al. 2011; Swanson et al. 2011). Final OTUs were then taxonomically classified using BLASTn against a curated database derived from GreenGenes, Ribosomal Database Project (RDPII) and NCBI (www.ncbi.nlm.nih.gov, (DeSantis et al. 2006), http://rdp.cme.msu.edu) and compiled into each taxonomic level. In this manuscript, *Prevotellaceae* in the context of genus taxa refers to unclassified members of this family at the genus level.

#### Data and statistical analysis

Alpha diversity or within-community diversity denotes the composite of organismal richness of a sample and the evenness of the organismal' abundance distribution and acts as a summary statistic of a single group (Morgan and Huttenhower 2012). Here, alpha diversity was defined by Shannon diversity indices (Hariri et al. 1990; Godden and Bajorath 2000; Claesson et al. 2010)**.** Beta diversity or between-community diversity indicates similarity (or difference) in organismal composition between samples and acts as a similarity (or dissimilarity) score between groups. Here, we show beta diversity using dendogram cluster analysis (first study) and dimension reduction ordination analysis (second study) (Morgan and Huttenhower 2012). Since in the work presented here we have compared whole microbiomes that are not dramatically distinct (e.g., in total sequence number), we have not normalized or rarefied sequencing counts to even sampling depth prior to determining diversity indices and richness (McMurdie and Holmes 2014).

Proportional abundance is reported in percentage and calculated as the number of sequences classified within each taxon normalized to total number of sequences in each sample). Dendograms were created with GelCompar software (BioNumerics). Principal component analysis was carried out using IBM (Portsmouth, U.K.) SPSS Statistics 21 for the 454 sequencing data obtained from the mouse study samples corresponding to the endpoint of the study (day 56) using the OTUs distribution at the genus and species level.

All statistical comparisons were performed using GraphPad Prism 6 for Windows (GraphPad Software, San Diego, CA). Unless otherwise stated, results are presented as means with standard deviations (SD). All data were tested by the D' Agostino and Pearson normality test for Gaussian distribution, where data did not fit the normal distribution this is indicated. Unless otherwise stated, between-group comparisons of more than two groups were done with one-way ANOVA with the Tukey's multiple comparisons test (for Gaussian distributed data) or with the Kruskal–Wallis test with the Dunn's multiple comparison test (non-Gaussian distributed data). Unless otherwise stated, within-group comparisons of more than two groups were performed using one-way repeated-measures ANOVA, with the Greenhouse–Geisser correction for nonsphericity, and the Tukey's multiple comparisons test with pairwise exclusion of missing data. For the few data that did not fit the normal distribution criteria, within-group comparisons of more than two groups were done with the Wilcoxon matched-pairs signed rank test. In all these comparisons of more than two diet groups/time-points, the *P*-values reported are multiplicity adjusted *P*-values (Wright 1992). Unless otherwise stated, comparisons between two groups were done with unpaired *t*-tests with Welch correction for nonequal variances where this was appropriate (i.e., depending on whether the *F* test to compare variances was significant). The significance level was set at *P* < 0.05.

## Results

For both studies, and reported elsewhere (Aslam et al. 2014; Powell et al. 2014), hemoglobin was significantly and similarly increased following the iron-repletion period with diets supplemented with Nano Fe(III) and FeSO_4_. Furthermore, weight gain in both iron groups was comparable, and comparable to the iron-sufficient reference group where there was one (i.e., in the mouse study) (Aslam et al. 2014; Powell et al. 2014). Apart from diet-induced iron deficiency, all animals were apparently healthy, with no visible gastrointestinal inflammation.

### First study

#### Fecal microbiota of Nano Fe(III)-supplemented rats appears similar in diversity to that of FeSO_4_-supplemented rats

**Figure 2:**
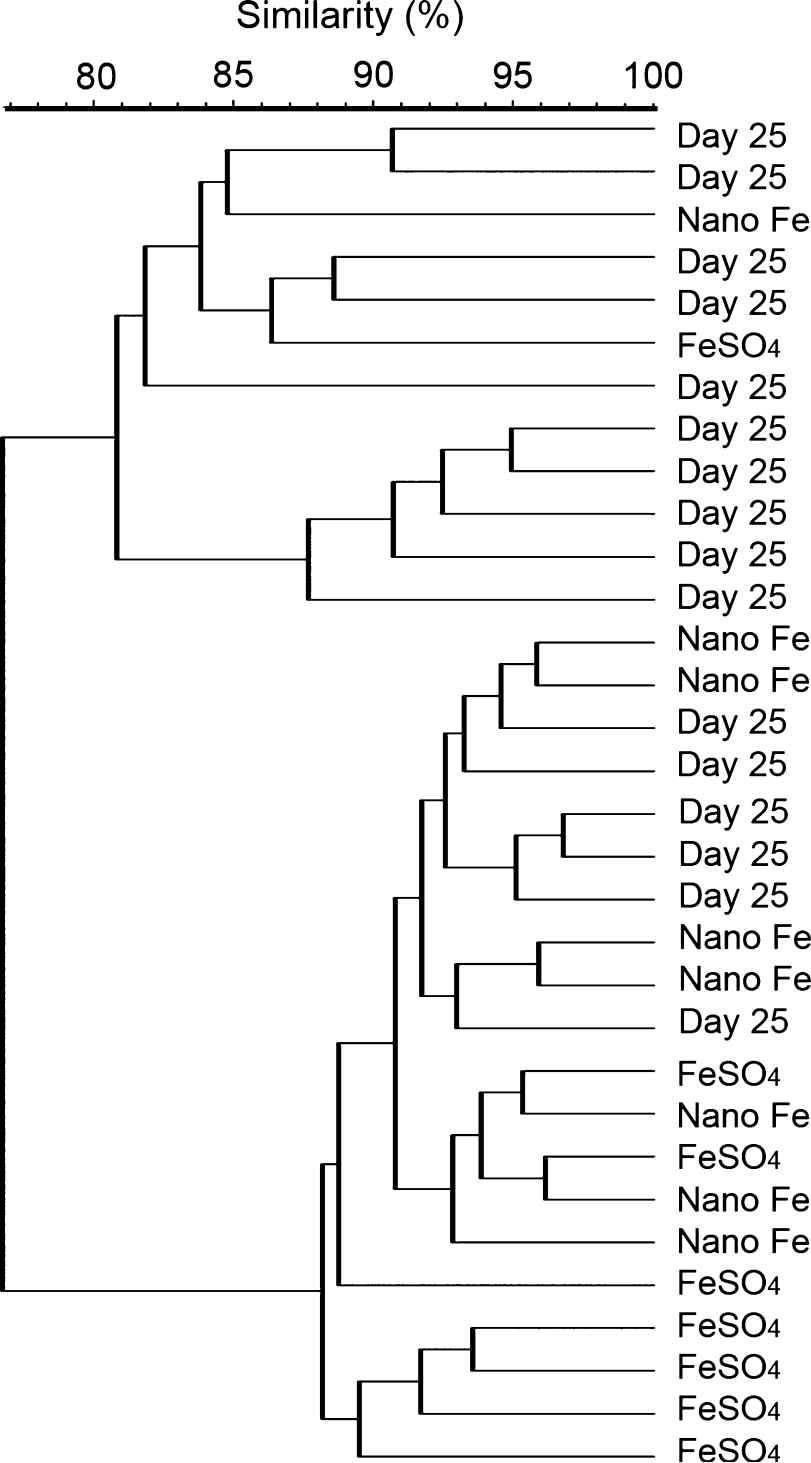
Dendrogram generated from DGGE profiles obtained from fecal samples of iron-deficient rats post Fe-depletion (day 25, *n* = 16) and 14 days following supplementation of the diet with either ferrous sulfate (FeSO_4_; *n* = 8) or tartrate-modified ferrihydrite (Nano Fe; *n* = 8) (day 39). Scale bar, percent similarity of profiles. Similarity coefficient used was Dice (with tolerance 1% and tolerance change 3%). Cluster matrix used was unweighted pair group method with arithmetic mean (UPGMA). DGGE, denaturing gradient gel electrophoresis.

In the first study, rats were made anemic via an initial dietary iron-depletion period of 25 days and this was followed by 14 days of iron repletion using the same iron-deficient diet but supplemented with FeSO_4_, Nano Fe(III), or unmodified synthetic ferrihydrite (Fh). The effect of dietary iron supplementation with either FeSO_4_ or Nano Fe(III) on the fecal microbiota of iron-deficient anemic rats was initially investigated using DGGE. Figure2 shows the between-community similarity (i.e., beta diversity) from the DGGE profiles at the end of the dietary iron-depletion period (day 25) followed by 14 days of dietary iron supplementation with either FeSO_4_ or Nano Fe(III) (day 39). Cluster analysis of the DGGE fingerprints did not show any obvious grouping of samples related to diet group and all samples showed at least 80% similarity. Table1 shows the within-community diversity (i.e., alpha diversity) computed from the DGGE fingerprints. Pairwise analysis of within-community diversity data showed that at the end of 14 days of dietary iron repletion with FeSO_4_ (i.e., day 39), rats showed significantly higher species richness (i.e., number of DGGE bands; *P* = 0.0008) and microbial diversity (i.e., Shannon indices, *P* ≤ 0.004) compared to the start of the FeSO_4_-supplementation period (i.e., day 25) (Table1). Conversely, in the animals allocated to the Nano Fe(III)-supplementation group there were no significant differences in any of the within-community diversity indices between the start and end of the iron supplementation period. However, it is important to note that the baseline diversity (i.e., day 25) in the Nano Fe(III) group was already high (*P* = 0.02 for the between-groups comparison) and an additional increase may not have been possible. This was, therefore, addressed further in study 2.

**Table 1:** Diversity data and indices computed from DGGE fingerprints of gut bacterial communities in iron-deficient rats on diets supplemented with two different Fe sources for 14 days

Group	Number of DGGE bands	Shannon diversity index (*H*′)^1^	Shannon evenness index (*E*_H_)^1^
FeSO_4_, *n* = 8			
After Fe-depletion (day 25)	33 (6)	3.4 (0.2)	0.986 (0.003)
After Fe-repletion^2^ (day 39)	54 (8)	3.9 (0.2)	0.992 (0.003)
*P*-value^3^	0.0008	0.0009	0.004
Nano Fe(III), *n* = 8			
After Fe-depletion (day 25)	42 (7)	3.7 (0.2)	0.992 (0.003)
After Fe-repletion^2^ (day 39)	49 (5)	3.9 (0.1)	0.994 (0.001)
*P*-value^3^	ns	ns	ns

Values are mean (SD). DGGE, denaturing gradient gel electrophoresis; FeSO_4_, ferrous sulfate; Nano Fe(III), nanoparticulate tartrate-modified ferrihydrite; ns, not statistically significant (*P* < 0.05 was considered significant).

1
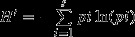
 and 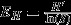; where *S* is the total number of different DGGE bands per sample and *pi*, is the proportional abundance of each band (i.e., the relative intensity of each band = intensity of each band divided by the total intensity of the bands obtained per sample) (Hariri et al. 1990; Godden and Bajorath 2000).

2 Fe-repletion period corresponding to the diets supplemented with either FeSO_4_ or Nano Fe(III).

3 Within-group analysis performed using paired two-tailed t-tests for comparison between day 25 and day 39 in each group.

While multivariate analysis, such as the DGGE cluster analysis presented above, is the best choice to evaluate overall differences in the microbial community structure, we additionally probed effects on specific bacterial groups using FISH. Total bacterial numbers at the end of the iron-depletion period were identical to those at the end of the iron-repletion period for all diet groups (Table2). The dominant bacterial groups (of those examined herein), in the gut microbiota of the rats throughout the study, were *Bacteroides/Prevotella* followed by *Lactobacillus/Enterococcus* (Table2). Between-group comparisons showed that the only significant difference between the diet groups was lower initial counts (i.e., prior to start of the intervention) of *Roseburia* in the rats allocated to the unmodified synthetic ferrihydrite-supplemented diet (Fh) compared to those allocated to the Nano Fe(III)-supplemented diet (*P* = 0.003). *Desulfovibrio* was the least abundant of the bacterial groups detected in this study (with levels often near or below the detection limit; Table2). Within-group comparisons showed significantly lower *Desulfovibrio* counts at the end of the study (i.e., day 39) for the iron-deficient group (*P* < 0.0001) and the FeSO_4_ group (*P* *=* 0.008); although this most likely was a reflection of higher starting values in animals allocated to these dietary groups. The same trend was observed for the unmodified synthetic ferrihydrite (Fh) group, although this did not reach statistical significance. FISH data showed no specific effect of the type of iron compound used to replete iron levels on the numbers of any of the bacterial groups investigated nor does it appear that these bacteria were the ones accountable for the lower within-community diversity observed following the initial iron-depletion period (Table1).

**Table 2:** Effect of dietary supplementation with different iron forms during 14 days upon population levels of marker bacteria as assessed by FISH

Group	Bacteria group							
	Total bacteria	*Bacteroides Prevotella*	Bifido	*Bacillus*	DSV	Lactob-enteroc	*Roseburia*	SFB
Fe-deficient								
Day 25	9.5 (0.6)	8.9 (0.3)	7.7 (0.6)	6.6 (0.6)	7.0 (0.3)	8.4 (0.2)	6.8 (1.0)	7.0 (0.8)
Day 39	9.6 (0.7)	9.1 (0.4)	7.1 (0.6)	6.8 (0.7)	6.0 (0.2)^2^	8.5 (0.6)	6.5 (0.8)	6.9 (0.6)
* P*-value^1^	ns	ns	ns	ns	<0.0001	ns	ns	ns
FeSO_4_								
Day 25	9.6 (0.4)	9.0 (0.4)	7.6 (0.4)	6.4 (0.6)	6.7 (0.6)	8.4 (0.3)	6.8 (0.7)	6.6 (0.6)
Day 39	9.4 (0.4)	8.8 (0.4)	7.3 (0.5)	6.8 (0.7)	6.0 (0.4)^2^	8.4 (0.2)	7.2 (0.8)	6.7 (0.8)
* P*-value^1^	ns	ns	ns	ns	0.008	ns	ns	ns
Fh								
Day 25	9.5 (0.4)	8.8 (0.3)	7.6 (0.6)	6.2 (0.5)	6.8 (0.7)	8.3 (0.4)	6.4 (0.4)	6.4 (0.7)
Day 39	9.3 (0.4)	8.6 (0.6)	7.3 (0.5)	7.0 (0.6)	6.1 (0.3)[Table-fn tf2-3]	8.3 (0.4)	6.5 (0.4)	6.6 (0.5)
* P*-value[Table-fn tf2-2]	ns	ns	ns	0.008	ns	ns	ns	ns
Nano Fe(III)								
Day 25	9.4 (0.4)	8.9 (0.4)	7.9 (0.6)	6.0 (0.4)	5.82 (0.07)[Table-fn tf2-3]	8.1 (0.3)	7.8 (0.6)	6.3 (0.6)
Day 39	9.4 (0.3)	8.9 (0.2)	7.2 (0.8)	6.4 (0.9)	6.1 (0.5)[Table-fn tf2-3]	8.1 (0.2)	7.6 (1.0)	6.3 (0.5)
* P*-value[Table-fn tf2-2]	ns	ns	0.04	ns	ns	ns	ns	ns

Values are mean counts (SD) of marker bacteria (Bacteria numbers are expressed as log10 counts/g wet feces for positively hybridized cells after in situ hybridization with group-specific probes or the universal probes for total bacteria (i.e., Eub388 I, II, and III).), *n* = 8 per group. In all groups, day 25 corresponds to after the Fe-depletion period, that is, following 25 days of iron-depletion with the Fe-deficient diet. For each group, day 39 corresponds to after the Fe-repletion period, that is, following 14 days on the Fe-deficient diet supplemented with each of the Fe materials; animals in the Fe-deficient group remained on the Fe-deficient diet throughout. FeSO_4_, ferrous sulfate; Fh, unmodified ferrihydrite; Nano Fe(III), nanoparticulate tartrate-modified ferrihydrite; FISH, fluorescence in situ hybridization; SD, standard deviation; Bifido, *Bifidobacterium*; DSV, *Desulfovibrio*; lactob-enteroc, *Lactobacillus/Enterococcus;* SFB, segmented filamentous bacteria or *Candidatus* Savagella; ns, not statistically significant (*P* < 0.05 was considered significant).

1 The majority of data passed the D' Agostino and Pearson normality test, and therefore within-group comparisons (i.e., day 25 versus day 39 for each diet group) were performed using paired two-tailed *t*-tests with pairwise exclusion of missing values. For the few data that did not fit the normal distribution criteria, within-group comparisons were done with the Wilcoxon matched-pairs signed rank test.Between-group comparisons (i.e., comparing the different diet groups at each timepoint) were done with one-way ANOVA with the Tukey's multiple comparisons test (for Gaussian distributed data) or with the Kruskal–Wallis test with the Dunn's multiple comparison test (non-Gaussian distributed data). The only significant difference for comparisons between-groups for day 25 or day 39 was found for *Roseburia* at day 25 between Fh and Nano Fe(III), *P* = 0.003 (multiplicity-adjusted value).

2 *Desulfovibrio* numbers were very close to the detection limit of FISH (<10^6^) for these groups. *Desulfovibrio* was not detectable in four of the samples in the Fh group on day 39 and in six of the samples in the Nano Fe(III) group on day 25.

Overall, data from the first study suggested that the initial iron-depletion period promoted lower within-community diversity in the microbiota and, that upon iron repletion with FeSO_4_, diversity significantly increased, but not with Nano Fe(III) supplementation (notwithstanding the caveat noted above on difference in baseline diversity). This was further investigated in the second study, this time also taking into consideration age and/or postweaning effects and introducing a longer period of dietary iron repletion to reduce the effect of short-term variations in the fecal microbiota caused by changing diets.

### Second study

#### Dietary iron depletion at weaning promotes lower within-community (alpha) microbial diversity in mice

We carried out a second study in mice, again, with an initial dietary iron-depletion period (28 days) followed by a longer iron-repletion period (28 days) with diets supplemented with either FeSO_4_ or Nano Fe(III). In this study, the effect of the dietary iron-depletion period on the fecal microbiota was investigated further alongside a reference group of mice that were iron-sufficient throughout the study to account for age or postweaning-related effects on the microbiota. Here, we investigated the entire fecal microbial community using 454-pyrosequencing of fecal 16S rRNA gene sequences. We have chosen 454-pyrosequencing in the second study rather than DGGE to examine microbial diversity since the phylogenetic identification of OTUs is more easily performed with this technique than with DGGE, which requires cloning and sequencing of excised bands. We obtained a range of 2613–6506 sequences per animal after quality control (Table3). Species richness and within-community microbial diversity (alpha diversity), as assessed by Shannon's diversity and evenness indices, were significantly lower following the iron-depletion period (i.e., comparisons between study days 0 and 28; *P* ≤ 0.0002), and also significantly lower than those observed in mice of the same age weaned onto the Fe-sufficient diet (*P* ≤ 0.03) (Table3). Throughout the rest of the study period (i.e., until day 56), the continuously Fe-deficient group showed significantly lower within-community microbial diversity than the continuously Fe-sufficient reference group (*P* < 0.0001).

**Table 3:** Diversity data and indices computed from 454-pyrosequencing data of gut bacterial communities in mice on diets supplemented with different Fe sources for 28 days

Group	Number of sequences	Richness (*S*)	Shannon diversity index (*H*′)^1^	Shannon evenness index (*E*_H_)^1^	Good's coverage (ESC %)^1^
Effect of Fe-depletion					
@ weaning (day 0), *n* = 23	3889 (746)	35 (4)^x^	2.4 (0.2)^x^	0.67 (0.06)	92 (2)
After Fe-depletion (day 28), *n* = 17	3592 (893)	27 (4)^y^	2.1 (0.2)^y^	0.63 (0.06)	93 (2)
Fe-sufficient (reference, day 28), *n* = 5	3439 (611)	37 (4)^x^	2.4 (0.2)^x^	0.66 (0.05)	91 (2)
Effect of Fe-repletion					
Fe-sufficient (reference) *n* = 5					
Day 28, *n* = 5	3439 (611)	37 (4)	2.4 (0.2)	0.66 (0.05)	91 (2)
Day 42, *n* = 5	3519 (1166)	34 (2)	2.3 (0.2)	0.66 (0.04)	91 (2)
Day 56, *n* = 5	2613 (446)^x^	43 (10)	2.6 (0.2)^x^	0.70 (0.04)^x^	87 (2)^x^
Fe-deficient (FD)					
Day 28, *n* = 5	3284 (717)^a^	25 (3)^a^	2.0 (0.2)	0.63 (0.06)	92 (3)
Day 42, *n* = 6	4057 (730)^a^	31 (3)^b^	1.8 (0.4)	0.51 (0.09)	94 (1)
Day 56, *n* = 6	6506 (1494)^b,y^	33 (3)^b^	1.7 (0.3)^y^	0.47 (0.09)^y^	95 (2)^y^
FeSO_4_					
Day 28, *n* = 4	3734 (443)	29 (4)	2.1 (0.2)	0.61 (0.05)	94 (2)
Day 42, *n* = 4	3853 (309)	25 (4)	2.0 (0.1)	0.64 (0.02)	93.7 (0.5)
Day 56, *n* = 4	3407 (1505)^xz^	32 (3)	2.1 (0.2)^z^	0.62 (0.05)^x^	89 (4)^x^
Nano Fe(III)					
Day 28, *n* = 8	3767 (1116)	28 (4)	2.1 (0.3)^a^	0.64 (0.08)^a^	93 (3)
Day 42, *n* = 8	5036 (1460)	31 (7)	2.0 (0.3)^a^	0.59 (0.06)^a^	94 (1)
Day 56, *n* = 7	4777 (911)^yz^	32 (9)	1.6 (0.1)^b,y^	0.47 (0.04)^b,y^	94 (1)^y^

Mice underwent an initial period of 28 days of dietary-induced Fe-depletion (i.e., weaning to study day 28), followed by 28 days of Fe-repletion (i.e., study day 28 to study day 56) with the Fe-deficient diet supplemented with FeSO_4_ or nano Fe(III) as per study outline in Figure1B. Data for the control Fe-deficient group (i.e., the group that remained on the Fe-deficient diet throughout) and the Fe-sufficient reference group (i.e., iron-replete mice) are also shown. Values are mean (SD). OTU, operational taxonomic unit; ESC, estimated sample coverage; d, day; FeSO_4_, ferrous sulfate; Nano Fe(III), nanoparticulate tartrate-modified ferrihydrite.

1
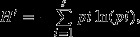
 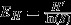, and 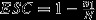; where *S*, is the total number of unique OTUs within each sample; *n1*, is the number of single copy OTU within each sample; *N,* is the total number of sequences, and *pi*, is the proportional abundance of each unique OTU within each sample (Hariri et al. 1990; Godden and Bajorath 2000; Claesson et al. 2010).

^a,b^Where statistical significance was established, means in a column within each group without a common superscripts letter differ: within-group comparisons (i.e., comparing the different timepoints for each diet group) were performed using one-way repeated-measures ANOVA, with the Greenhouse–Geisser correction for nonsphericity and the Tukey's multiple comparisons test with pairwise exclusion of missing data. *P* < 0.05 was considered significant (multiplicity adjusted).

^x,y,z^Between-group comparisons (i.e., comparing the different diet groups) at the end of the study (day 56) or comparing the effect of Fe-depletion (day 0 versus day 28) was carried out with ordinary one-way ANOVA with the Tukey's multiple comparison test: where statistical significance was established, means without a common superscripts letter differ. *P* < 0.05 was considered significant (multiplicity adjusted).

**Figure 3:**
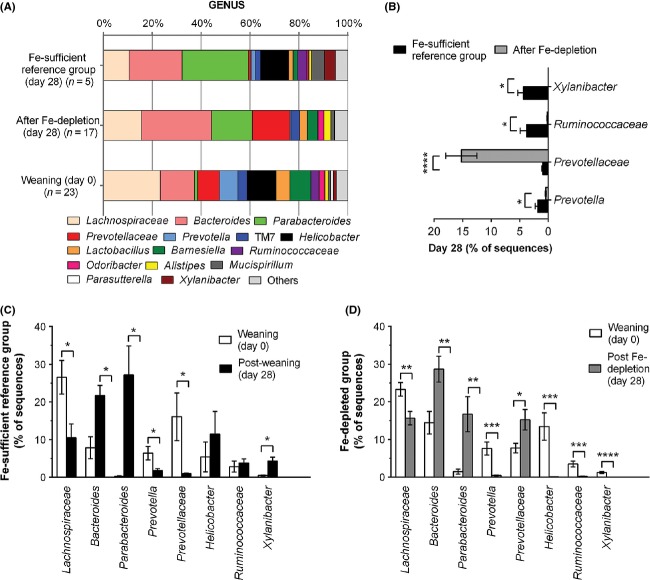
Fecal microbiota of mice before (weaning, day 0) and after 28 days of dietary-induced Fe-depletion (day 28). (A) Data shown as mean percentage of sequences for the most abundant bacteria genus as determined by 454-pyrosequencing. TM7, candidate division TM7 (Hugenholtz et al. 2001). Data for the Fe-sufficient reference group (i.e., mice that were iron replete throughout) are also shown. (B) Data shown as mean (±SEM) for the proportions of each genus for which there were statistically significant alterations 28 days post-weaning onto the Fe-deficient diet (*n* = 17) in comparison with mice of the same age weaned onto the reference Fe-sufficient diet (*n* = 5). (C and D) Age-related statistically significant changes for the proportions of each genus (mean ± SEM) in the (C) Fe-sufficient reference group (*n* = 5) and the (D) Fe-depleted group (*n* = 17). *P ≤ 0.04; **P ≤ 0.005; ***P ≤ 0.0009; ****P < 0.0001 using unpaired *t*-tests. *Prevotellaceae* refers to unclassified members of this family at the genus level.

We further investigated the effect of diet-induced Fe-depletion on the fecal microbial diversity in mice by looking at the proportion of each genus at the start and at the end of the 28 days Fe-depletion period, and comparing these with data for mice of the same age in the continuously Fe-sufficient reference group (Fig.3A). Comparison of the proportions for each bacterial genus in mice that underwent 28 days of Fe-depletion post-weaning (*n* = 17), in relation to mice in the continuously Fe-sufficient reference diet (*n* = 5), showed that *Prevotella* (*P* = 0.04), *Ruminococcaceae* (*P* = 0.03) and *Xylanibacter* (*P* = 0.01) all decreased significantly with the Fe-depletion, whereas unclassified members at the genus level of *Prevotellaceae* increased markedly (*P* < 0.0001) (Fig.3B). Helicobacter was also considerably higher in the Fe-sufficient reference group (11 ± 6%) when compared to the Fe-deficient diet group (0.11 ± 0.02) but this did not quite reach statistical significance when correcting for the different mouse numbers in both groups (*P* = 0.1, unpaired *t*-test with Welch's correction). Eight bacterial genera were significantly affected following weaning onto the Fe-deficient diet (day 0 versus day 28, Fig.3D) but the observed changes in *Lachnospiraceae*, *Bacteroides*, *Parabacteroides*, and to some extent *Helicobacter*, were comparable to the age/weaning changes observed in the Fe-sufficient reference group (Fig.3C).

#### Iron repletion with Nano Fe(III) generates lower alpha microbial diversity in mice than iron repletion with FeSO_4_

Following the initial Fe-depletion period, supplementation of the diet with Nano Fe(III) for a further 28 days generated similar alpha microbial diversity (i.e., within-community) to mice kept on the Fe-deficient diet throughout the 56 study days, and this was significantly lower than for the FeSO_4_ (*P* = 0.004) and the Fe-sufficient reference (*P* < 0.0001) groups (Table3). Conversely, at the end of the study, FeSO_4_-supplemented mice displayed fecal alpha microbial diversity indices significantly higher than those in the Fe-deficient group (*P* = 0.006) and closer to the Fe-sufficient reference group (*P* = 0.01) (Table3).

#### Dietary iron supplementation only partially restores beta microbial diversity in iron-depleted mice, irrespective of the iron source

**Figure 4:**
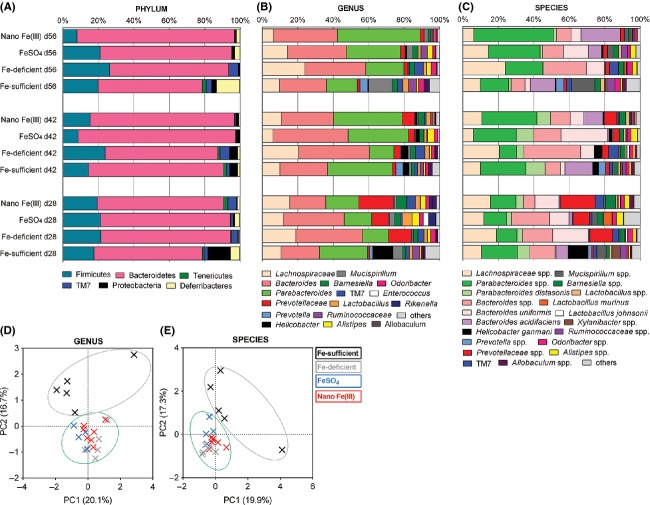
Fecal microbial diversity of mice on diets supplemented with Nano Fe(III) or FeSO_4_ during 28 days. Data shown as mean percentage of sequences determined by 454-pyrosequencing for each diet group at each time-point during the 28 days Fe-repletion period (i.e., study day 28 to study day 56) for: (A) phylum, (B) 98% most predominant genus and (C) 98% most predominant bacterial species. TM7, candidate division TM7 (Hugenholtz et al. 2001); FeSO_4_, ferrous sulfate; Nano Fe(III), tartrate-modified ferrihydrite. *Prevotellaceae* refers to unclassified members of this family at the genus level. Score plot of the principal component analysis of variance based on the normalized abundance of each bacterial genus (D) and species (E). Percentage variance values accounted for by the two first components (PC1 and PC2) are reported in parenthesis. The different diet groups as defined in Figure1B are color coded in black (Fe-sufficient reference diet throughout), gray (Fe-deficient diet throughout), blue (FeSO_4_-supplemented diet), and red (Nano Fe(III)-supplemented diet).

Figure4 presents OTU relative abundances at the phylum, genus, and species level during the 28 days of iron repletion (i.e., from study day 28 to day 56) with either Nano Fe(III) or FeSO_4_, alongside data for mice kept continuously in the Fe-sufficient reference diet and mice kept continuously in the Fe-deficient diet for the entire study period. *Bacteroides* and *Parabacteroides* were the two most dominant genera in the core fecal microbiota of mice before and after iron supplementation with either Nano Fe(III) or FeSO_4_ (Fig.4). Exploration of beta microbial diversity (i.e., between-community), with principal components analysis of microbial genera and species data at the end of the study (day 56), suggested two main clusters: one for the continuously Fe-sufficient reference group and the other for all three groups which underwent a period of iron depletion (i.e., Fe-deficient, FeSO_4_, and Nano Fe(III) diet groups) (Fig.4 D and E). These data indicated that the initial period of Fe-depletion at the time of weaning imprints lower beta diversity in the microbiota that is not recovered with dietary iron repletion, irrespective of the iron source.

**Figure 5:**
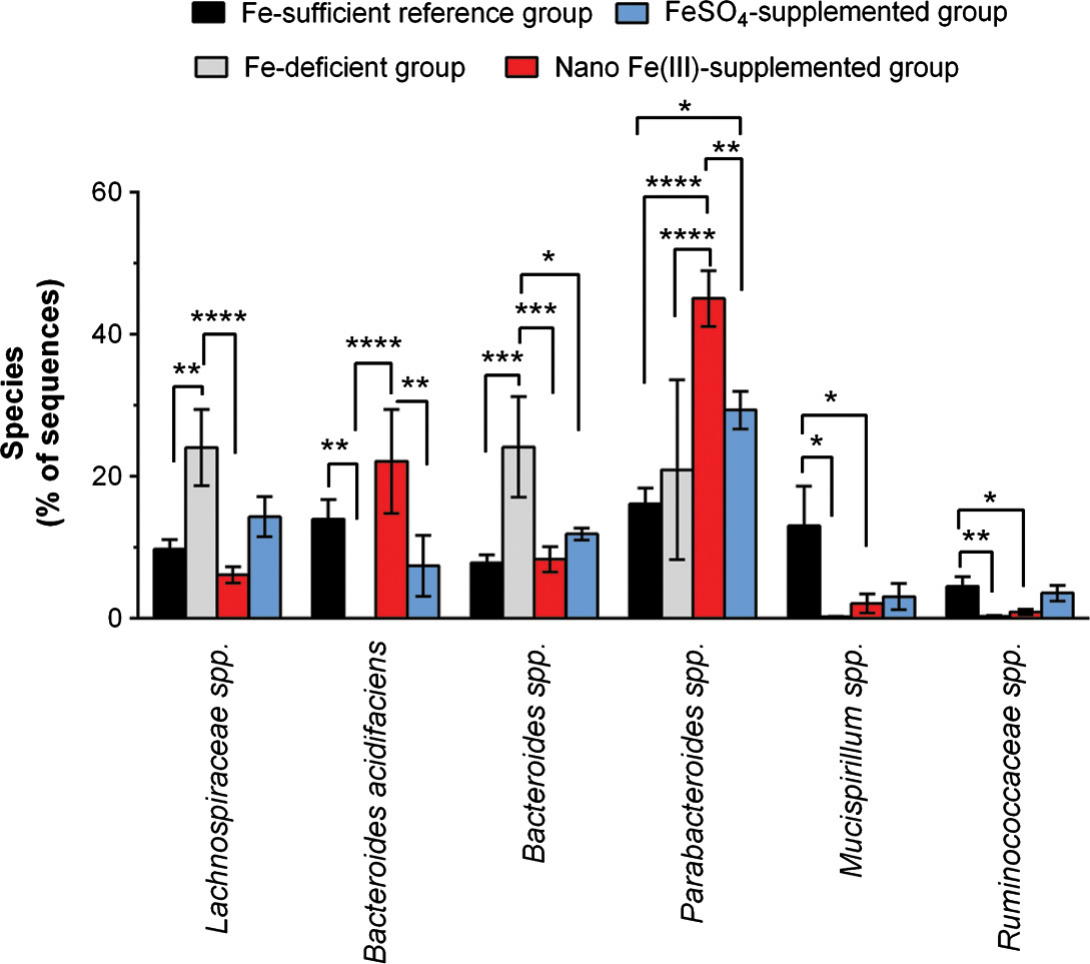
Changes in representative bacterial species of the fecal microbiota of mice following 28 days of diets supplemented with Nano Fe(III) or FeSO_4_. Data shown as mean (SEM) percentage of sequences determined by 454-pyrosequencing for each bacterial species for which there were statistically significant alterations at the end of the Fe-repletion period. Data for the Fe-sufficient reference group (i.e., mice fed the regular Fe-sufficient diet throughout the study period) and the Fe-deficient control group (i.e., mice maintained on the Fe-deficient diet throughout the study) are shown at the same timepoint (study day 56). Numbers in each diet group are as follows: *n* = 5, Fe-sufficient reference group (black); *n* = 6, Fe-deficient control group (gray); *n* = 4, FeSO_4_ group (blue); *n* = 8, Nano Fe(III) group (red). Between-group comparisons (i.e., comparisons between the four different diets at day 56) were done with repeated-measures two-way ANOVA with the Tukey's multiple comparison test. *P *≤* 0.04; **P ≤ 0.007; ***P ≤ 0.0009; ****P < 0.0001 (all multiplicity-adjusted *P*-values).

We also considered whether individual species were affected following the 28 days of the Fe-repletion period with the Nano Fe(III)- or FeSO_4_-supplemented diets in relation to the Fe-sufficient and the Fe-deficient groups. At the end of the study, the main differences were again observed in the iron depleted mice (i.e., Fe-deficient diet group), which showed significantly higher *Lachnospiraceae* spp. (*P* ≤ 0.005) and *Bacteroides* spp. (*P* ≤ 0.04), and lower *Bacteroides acidifaciens* (*P* ≤ 0.007), *Mucispirillum* spp. (*P* ≤ 0.04), and *Ruminococcaceae* spp. (*P* ≤ 0.02) than mice in the other diet groups. Between-group comparisons at the end of the iron-repletion period for the Nano Fe(III)-supplemented group in relation to the FeSO4-supplemented group showed significant differences in *Parabacteroides* spp. (*P* = 0.003) and *Bacteroides acidifaciens,* (*P* = 0.005), which were both higher in the Nano Fe(III) group, and a tendency for lower sequences of *Lachnospiraceae* spp*. and Bacteroides* spp. (Fig.5).

## Discussion

We have previously reported on the intestinal uptake mechanism of a nanoparticulate tartrate-modified ferrihydrite (here termed Nano Fe(III)) that we believe mimics the result of the digestive processes with dietary iron (Pereira et al. 2013). We have reported, separately, on its physico-chemical characterization and equivalent bioavailability to FeSO_4_ (Powell et al. 2014).

Generally, the complex of bacteria in the gut is stable in healthy individuals (Arumugam et al. 2011; Yatsunenko et al. 2012), but, one of the anxieties surrounding engineered nanoparticulate compounds that are added to the diet is their potential to induce changes to the microbiome (Sawosz et al. 2007; Han et al. 2010; Pineda et al. 2012; Wang et al. 2012). Moreover, soluble oral iron has been associated with detrimental changes to the gut microbiota in gastrointestinal disease (Zimmermann et al. 2010; Werner et al. 2011)and with increased growth and adhesion of enteric pathogens such as *Salmonella* (Kortman et al. 2012).

However, when investigating a complex microbiota, such as that of the gut, concentrating on individual genera or species has limitations, as this can be misrepresentative of the true impact within the diverse microbiome and, therefore, here we sought to also evaluate effects on overall microbial diversity and considered both alpha and beta diversity outputs (Swenson 2011; Conlan et al. 2012; Morgan and Huttenhower 2012). These two outputs are complementary in that they reveal different aspects of the microbial community structure: alpha diversity is a snapshot statistic of how many different OTUs are within a sample/group and of how evenly distributed they are, whereas beta diversity is more representative of how the different OTUs are distributed between samples or groups of samples. There is only one report with oral iron and the microbiota where Werner et al. (2011) have attempted to characterize effects on the overall gut microbial diversity in mice. These authors have reported that supplementation of the diet with FeSO_4_ in a mouse model of Crohn's disease does not significantly alter the within-community (alpha) microbial diversity but does change the between-community (beta) diversity in comparison to mice that were luminally iron depleted (Werner et al. 2011). This previous work was carried out in a diseased model and here we wanted to investigate the effects of supplementing the diets of anemic but otherwise healthy animals with Nano Fe(III) as opposed to FeSO_4_. Our rationale is that gut bacteria do not possess endocytic mechanisms to take up nanoparticles, (Jermy 2010) and therefore should not be able to utilize the iron from Nano Fe(III) to the same extent as iron from soluble Fe sources. Indeed, it has been reported previously that FeSO_4_ has a more pronounced effect on some bacterial groups of the gut microbiota than poorly soluble iron sources such as electrolytic iron (Dostal et al. 2012). And our own preliminary data suggest a promoting effect of Nano Fe(III) on beneficial bacteria of the gut microbiota in severely anemic rats with diarrhea (Pereira et al. 2014). Notably, from our DGGE fingerprinting data there was no obvious clustering (between-community diversity) (Fig.2) or any significant differences in the within-community microbial diversity between the FeSO_4_-and the Nano Fe(III)-supplemented diet groups at the end of the 14 days of iron supplementation (Table1). Further investigation using FISH confirmed these findings as it showed no significant differences between FeSO_4_- or Nano Fe(III)-supplemented diet groups at the end of the study (Table2). In our study, no significant differences were observed in *Desulfovibrio* numbers, which were all low, between the diets supplemented with iron from the different sources (soluble FeSO_4_, nanoparticulate Nano Fe(III), and poorly soluble Fh), or the diet not supplemented with Fe. This is opposed to what was observed by Werner et al. (2011) in a Crohn's disease mouse model where small but significant increases in *Desulfovibrio* were observed in the animals supplemented with FeSO_4_, which supports the fact that the *Desulfovibrio* genus assumes most importance in a diseased colon, as previously reported (Gibson et al. 1991; Marquet et al. 2009; Rowan et al. 2009). Nonetheless, our DGGE data suggest that following dietary iron depletion there is a lower within-community microbial diversity in comparison to the period following iron supplementation (particularly with FeSO_4_), however, the main limitation of this first study is the lack of a continuously Fe-sufficient reference group to account for age or weaning-related changes to the microbiota (Ottman et al. 2012). This group was, therefore, incorporated in the design of the second study, alongside a longer period of dietary iron repletion to ensure that short-term variability in the fecal microbiota caused by changing diets is reduced (David et al. 2014).

Our second study largely confirms the preliminary findings from the first study in that there are no marked differences in microbiome composition in mice supplemented with Nano Fe(III) compared to mice supplemented with FeSO_4_. We do acknowledge that the two studies used different methodologies but both approaches lead to similar conclusions. This strongly suggests that in otherwise healthy iron-deficient rodents there is no detrimental impact on the gut microbiome from supplementing the diet with FeSO_4_ or nano Fe(III).

Most interestingly, in this second study, mice weaned onto the Fe-deficient diet and kept on the same diet for the entire 8 weeks of the study showed significantly lower within-community microbial diversity than mice weaned onto the Fe-sufficient reference diet (Table3). It is commonly recognized that the complex gut microbiome develops fully following introduction of solid foods at weaning (Ottman et al. 2012) and our data suggest that oral iron may be a limiting nutrient in the process. Interestingly, despite having similar systemic bioavailability to FeSO_4_ (Aslam et al. 2014; Powell et al. 2014), Nano Fe(III) supplementation did not increase the within-community microbial diversity to the same extent as FeSO_4_ (Table3). We do acknowledge that these differences may be considered small, but we would not expect large changes following a dietary intervention where only the iron source is different and, therefore, we still consider these small differences between diet groups to be important. Mice weaned onto the Fe-sufficient diet and maintained on the same diet throughout the study also showed significantly different between -community microbial diversity, assessed with ordination analysis of genera and species data, than mice that were weaned onto the Fe-deficient diet, and this was not affected by the subsequent period of dietary iron repletion. We do appreciate that assessing beta diversity using taxa data may be subjected to classification biases, even though we tried to minimize these by using the curated combined gene database and tools. Consequently, we have conducted the ordination analysis based on unclassified OTU data and this mostly confirms our findings while allowing for a better separation of the mice that were supplemented with oral iron following the initial iron-depletion period (Fig. S1).

This finding agrees with our hypothesis that iron from nanoparticulate compounds such as Nano Fe(III) is less available to gut bacteria in comparison to soluble iron, in a similar fashion to what has been reported for poorly soluble electrolytic iron (Dostal et al. 2012). But this hypothesis needs to be further investigated in pure and mixed bacterial cultures.

With the caveats mentioned above, individual taxa analysis from 454-pyrosequencing data 28-days post-weaning suggested that *Helicobacter, Ruminococcaceae*, and Xylanibacter spp. numbers are significantly reduced by dietary iron depletion, whereas *Prevotellaceae* spp. increase in numbers (Fig.3B). Moreover, the small differences in fecal microbial diversity in mice supplemented with Nano Fe(III) in relation to those supplemented with FeSO_4_ appeared particularly driven by changes in numbers of the dominant bacteria groups, namely those of *Parabacteroides*, *Lachnospiraceae*, *and Bacteroides* species (Fig.5). The overall significant increase in *Prevotellaceae* with iron depletion after weaning (Fig.3B) could be related to the typical effect of enterotypes described before in mice (Hildebrand et al. 2013) and humans (Arumugam et al. 2011; Wu et al. 2011), and in our study this increase does not appear to be at the expense of *Bacteroides* but rather at the expense of *Ruminococcaceae* and *Helicobacter*. Interestingly, the Prevotella enterotype is usually associated with vegetarian diets rich in fiber and carbohydrates, whereas the Bacteroides enterotype is associated with diets rich in animal protein (Wu et al. 2011), maybe the different availability of iron from both types of diet (Engelmann et al. 1998; Etcheverry et al. 2006) plays a role in defining these preferred arrangements within the gut microbiome.

In our study, iron depletion did not promote the decrease in overall *Bacteroides* genus numbers reported in another study (Dostal et al. 2012), but it did promote significantly lower *Bacteroides acidifaciens* numbers in relation to the iron-replete reference group (Figs.4C and 5). Nonetheless, the absence of a reference group to account for age-related changes in the study by Dostal et al. makes interpretation of their data challenging. Furthermore, in the study by Dostal et al. the probe used to enumerate *Bacteroides* spp. could have cross-reacted with species belonging to *Prevotella* and *Prevotellaceae*, which are closely related and belong to the *Bacteroidetes* class.

## Conclusion

Taken together our data evidence the foremost impact of reducing luminal iron levels during weaning on the gut microbiota of rodents. We show that animals that are weaned onto an Fe-deficient diet had significantly lower fecal microbial diversity than animals weaned onto an Fe-sufficient reference diet. Furthermore, we show that upon dietary iron repletion with bioavailable iron sources the microbial diversity is only partially restored and, more so with FeSO_4_ than with nanoparticulate Fe(III). This finding suggests that Nano Fe(III) may be less available to some gut bacteria and this may present an advantage in terms of oral iron repletion in gastrointestinal disease and enteropathogenic infection. Further studies in humans and animal models are now warranted.

## Data Accessibility

All data sets are archived at MRC HNR and together with raw sequence files can be made available upon request by the corresponding author.

## Supporting Information

**Figure S1.** Score plot of the principal component analysis of variance based on the abundance of each OTU. Percentage variance values accounted for by the two first components (PC1 and PC2) are reported in parenthesis. The different diet groups are color coded in black (Fe-sufficient reference diet throughout), gray (Fe-deficient diet throughout), blue (FeSO4-supplemented diet), and red (Nano Fe(III)-supplemented diet).

**Table S1.** Composition of the modified AIN-93G purified rodent diet (Reeves et al.).
