# Supplementary material for: Dietary iron depletion at weaning imprints low microbiome diversity and this is not recovered with oral nano Fe(III)
Source: Microbiologyopen. 2014 Dec 2;4(1):12–27. doi: 10.1002/mbo3.213 (PMC4335973; doi:10.1002/mbo3.213)
Supplement: Supplementary file 1 [file mbo30004-0012-sd1.pdf]

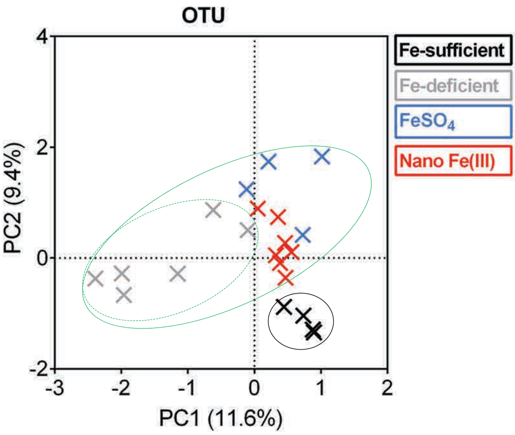

Supplementary Figure S1. Score plot of the principal component analysis of variance based on the abundance of each OTU. Percentage variance values accounted for by the two first components (PC1 and PC2) are reported in parenthesis. The different diet groups are colour coded in black (Fe-sufficient reference diet throughout), grey (Fe-deficient diet throughout), blue ($FeSO_4$-supplemented diet) and red (Nano Fe(III)-supplemented diet).
