# Supplementary material for: Dietary iron depletion at weaning imprints low microbiome diversity and this is not recovered with oral nano Fe(III)
Source: Microbiologyopen. 2014 Dec 2;4(1):12–27. doi: 10.1002/mbo3.213 (PMC4335973; doi:10.1002/mbo3.213)
Supplement: Supplementary file 2 [file mbo30004-0012-sd2.pdf]

Dietary iron depletion at weaning imprints low microbiome diversity and this is not recovered with oral nano Fe(III).

Pereira DIA, Aslam MF, Frazer DM, Schmidt A, Walton GE, McCartney AL, Gibson GR, Anderson GJ, Powell JJ

Supplementary Table S1. Composition of the modified AIN-93G purified rodent diet (Reeves et al 1993).

Ingredient	g/kg_{diet}	Ingredient	g/kg_{diet}	Ingredient	g/kg_{diet}
Casein	200	Folic acid	0.002	Fe [1]	
Sucrose	117.6	Biotin	0.0002	Zn	0.03
Cornstarch	397.5	Cyanocoba-lamin (0.1%)	0.025	Mn	0.01
Dyetrose	132	Vitamin A Palmitate	4000 IU/kg_{diet}	Cu	0.006
L-cysteine	3	Vitamin E Acetate	75 IU/kg_{diet}	I	0.0002
Cellulose	50	Vitamin D3	1000 IU/kg_{diet}	Mo	0.00015
Soybean Oil	70	Vitamin K1	0.00075	Se	0.00015
t-Butylhydroquinone	0.014	Ca	5	Si	0.005
Choline Bitartrate	2.5	P	1.561	Cr	0.001
Thiamin HCl	0.006	K	3.6	F	0.001
Riboflavin	0.006	S	0.3	Ni	0.0005
Pyridoxine HCl	0.007	Na	1.019	B	0.0005
Niacin	0.03	Cl	1.571	Li	0.0001
Ca panthothenate	0.016	Mg	0.507	V	0.0001

[1]The iron content of each diet was analysed by inductively-coupled plasma optical emission spectrometry as reported previously (Aslam et al In Press, Powell et al 2014). <u>Rat study</u>: Fe-deficient diet 3.1 (0.6) mgFe/kg diet; $FeSO_4$-supplemented diet 35.8 (0.1) mgFe/kg diet; Nano Fe(III)-supplemented diet 35.7 (0.1) mgFe/kg diet; unmodified Fh-supplemented diet 31.4 (0.5) mgFe/kg diet. <u>Mouse study</u>: Fe-deficient diet 1.0 (0.2) mgFe/kg diet; $FeSO_4$-supplemented diet 19.4 (0.4) mgFe/kg diet; Nano Fe(III)-supplemented diet 22.9 (0.1) mgFe/kg diet; control Fe-sufficient diet 43.1 (0.1) mgFe/kg diet (added as ferric citrate).

Supplementary Table S2. Oligonucleotide probes and hybridisation conditions used in FISH analysis of faecal bacteria.

Probe	Target Bacterial group	Sequence (5'→ 3')	Hybridisation temperature (°C)	References
**Eub338- I** [1]	domain bacteria	GCTGCCTCCCGTAGGAGT	46	(Daims et al 1999)
**Eub338-II** [1]	domain bacteria	GCAGCCACCCGTAGGTGT	46	(Daims et al 1999)
**Eub338-III** [1]	domain bacteria	GCTGCCACCCGTAGGTGT	46	(Daims et al 1999)
**Bac303**	*Bacteroides - Prevotella*	CCAATGTGGGGGACCTT	46	(Manz et al 1996)
**Bif164** [2]	*Bifidobacterium*	CATCCGGCATTACCACCC	50	(Langendijk et al 1995)
**Bmy843**	*Bacillus*	CTTCAGCACTCAGGTTCG	50	(Salzman et al 2002)
**Dsv687**	*Desulfovibrio*	TACGGATTTCACTCCT	48	(Ramsing et al 1996)
**Lab158** [2]	*Lactobacillus-Enterococcus*	GGTATTAGCAYCTGTTTCCA	50	(Harmsen et al 1999)
**Rrec584**	*Roseburia*	TCAGACTTGCCGYACCGC	50	(Aminov et al 2006, Walker et al 2005)
**SFB1008**	segmented filamentous bacteria	GCGAGCTTCCCTCATTACA AGG	50	(Snel et al 1995)

Oligonucleotide probes were commercially synthesized and 5'-labeled with the fluorescent dye Cy3 (Sigma-Aldrich).

[1] These probes were used combined at equal concentrations (50 ng/µl) and 35 %(w/v) formamide was added to hybridisation buffer.

[2] Samples were pre-treated with lysosome at 1 mg/ml of 50,000 Units (U)/mg protein prior to hybridisation.

Supplementary References

Aminov RI, Walker AW, Duncan SH, Harmsen HJM, Welling GW, Flint HJ (2006). Molecular diversity, cultivation, and improved detection by fluorescent in situ hybridization of a dominant group of human gut bacteria related to Roseburia spp. or Eubacterium rectale. *Appl Environ Microbiol* **72:** 6371-6376.

Aslam MF, Frazer DM, Faria N, Bruggraber SFA, Wilkins SJ, Mirciov C *et al* (In Press). Ferroportin mediates the intestinal absorption of iron from a nanoparticulate ferritin core mimetic in mice. *FASEB J*.

Daims H, Bruhl A, Amann R, Schleifer KH, Wagner M (1999). The domain-specific probe EUB338 is insufficient for the detection of all Bacteria: Development and evaluation of a more comprehensive probe set. *Systematic and Applied Microbiology* **22:** 434-444.

Harmsen HJM, Elfferich P, Schut F, Welling G (1999). A 16S rRNA-targeted Probe for Detection of Lactobacilli and Enterococci in Faecal Samples by Fluorescent In Situ Hybridization. *Microbial Ecology in Health and Disease* **11:** 3-12.

Langendijk PS, Schut F, Jansen GJ, Raangs GC, Kamphuis GR, Wilkinson MHF *et al* (1995). Quantitative Fluorescence in-Situ Hybridization of Bifidobacterium Spp with Genus-Specific 16s Ribosomal-Rna-Targeted Probes and Its Application in Fecal Samples. *Appl Environ Microbiol* **61:** 3069-3075.

Manz W, Amann R, Ludwig W, Vancanneyt M, Schleifer KH (1996). Application of a suite of 16S rRNA-specific oligonucleotide probes designed to investigate bacteria of the phylum cytophaga-flavobacter-bacteroides in the natural environment. *Microbiology-Uk* **142:** 1097-1106.

Powell JJ, Bruggraber SF, Faria N, Poots LK, Hondow N, Pennycook TJ *et al* (2014). A nano-disperse ferritin-core mimetic that efficiently corrects anaemia without luminal iron redox activity. *Nanomedicine: Nanotechnology, Biology and Medicine*.

Ramsing NB, Fossing H, Ferdelman TG, Andersen F, Thamdrup B (1996). Distribution of bacterial populations in a stratified fjord (Mariager Fjord, Denmark) quantified by in situ hybridization and related to chemical gradients in the water column. *Appl Environ Microbiol* **62:** 1391-1404.

Reeves PG, Nielsen FH, Fahey GC, Jr. (1993). AIN-93 purified diets for laboratory rodents: final report of the American Institute of Nutrition ad hoc writing committee on the reformulation of the AIN-76A rodent diet. *J Nutr* **123:** 1939-1951.

Salzman NH, de Jong H, Paterson Y, Harmsen HJM, Welling GW, Bos NA (2002). Analysis of 16S libraries of mouse gastrointestinal microflora reveals a large new group of mouse intestinal bacteria. *Microbiology-Sgm* **148:** 3651-3660.

Snel J, Heinen PP, Blok HJ, Carman RJ, Duncan AJ, Allen PC *et al* (1995). Comparison of 16S rRNA sequences of segmented filamentous bacteria isolated from mice, rats, and chickens and proposal of "Candidatus Arthromitus". *Int J Syst Bacteriol* **45:** 780-782.

Walker AW, Duncan SH, McWilliam Leitch EC, Child MW, Flint HJ (2005). pH and peptide supply can radically alter bacterial populations and short-chain fatty acid ratios within microbial communities from the human colon. *Appl Environ Microbiol* **71:** 3692-3700.
